# Supplementary material for: Association of middle cerebral artery aneurysms and variation of the A1 segment
Source: PLoS One. 2025 Mar 25;20(3):e0319500. doi: 10.1371/journal.pone.0319500 (PMC11936263; doi:10.1371/journal.pone.0319500)
Supplement: Attachment — Submitted filename: Response to Reviewers.docx [file pone.0319500.s001.docx]

Dear Editors,

On behalf of my co-authors, we thank you for giving us the opportunity to revise and improve the quality of our article. We have carefully reviewed the comments from both the reviewers and yourselves, and have made revisions which are marked in red within the paper. We have made every effort to address the comments provided. Please find attached the revised version, which we would like to submit for your kind consideration.

In the process of revision, we added 3 references, converted a table into a graph, and included an additional graph, resulting in a total of 55 references, 8 figures, and 4 tables. The corresponding text has undergone significant changes, and the original column order has been greatly modified.

During the revision process, we enlisted the help of a native English speaker with a research background to review our manuscript. If you identify any issues, please do not hesitate to bring them to our attention, and we will seek assistance from professional organizations to improve the language quality.

We have prepared a detailed response letter addressing each of the reviewer comments, which can be found at the end of this letter. We found the comments to be extremely helpful and would like to express our gratitude to both you and the reviewers for your valuable feedback.

In addition, our colleague Dr. Yue has recently changed her name. Therefore, I will update her name from Yanxiang Yue to Xi Yue.

We hope that this revised version meets the standards for publication in your journal. Thank you for considering our submission, and we look forward to hearing from you soon.

 Sincerely,

Hongzhi Gao

**Reviews’ Comments to the Author**

**Reviewer 1:**

There are certain editions, which I would suggest prior to accepting the manuscript in its current form.
1. The authors must describe their methodology in detail- in regards to reason for exclusion of each section as per their flow diagram. This reasoning can be added to the discussion section.

Author response:Thank you for your opinion.We have revised the flow chart to make it more concise and clear, and we have incorporated a description of it in the discussion section as per your feedback in Line200-210.

2. Some demographic results are duplicated in the description. These need to be removed. Page 7 line 129-139. The authors can focus on describing positive highlights from these results table in description.

Author response:Thank you for your opinion. Duplicates in the population data description have been removed.

3. Table 4 and 5 must include the numerical values and percentages of parameters compared between the two groups. Similarly in other statistical tables.
Author response: Figures 4 and 5 have been updated and corrected.
Decision: Minor revision

Author response:Thank you!

**Reviewer 2:**

As a proficient writer, I appreciate your thorough evaluation of the relationship between vascular variation and aneurysm rupture, as well as its subsequent exacerbation. However, I noticed that you did not provide a comprehensive description of how vascular variation is measured; this aspect could be elaborated upon. Furthermore, regarding the final discussion on mechanisms, there has been insufficient exploration of the abnormalities in various blood vessels and their role in aneurysm pathogenesis. Potential mechanisms such as inflammation, immune responses, mutations, or alterations in cerebral vascular circulation warrant further consideration for a more nuanced explanation.

Author response: The method of measuring artery diameter is supplemented in lines 115-118. The discussion on the role of vascular abnormalities in the pathogenesis of aneurysms is described in lines 235-264, and detailed supplements have been made according to your comments.

**Reviewer 3:**

The study investigates the relationship between variations in the circle of Willis and middle cerebral artery (MCA) aneurysms.
It shows higher prevalence in the aneurysm group of A1 dysplasia in the affected side, smaller average diameter of middle cerebral arteries, MCA diameter on the affected side smaller in the A1 dysplasia group (2.156 ± 0.5256mm) compared to the A1 normal development group (2.405 ± 0.5718mm, P = 0.0114). Furthermore, the average maximum aneurysm diameter was larger in the A1 dysplasia group. The presence of ipsilateral A1 hypoplasia had a statistically
significant effect on the occurrence and development of MCA aneurysms.
The study therefore suggests that the variation in the circle of Willis may impact the occurrence and progression of MCA aneurysms by altering blood flow distribution, constricting the diameter of the parent artery, and shifting the location of blood flow impact.
The study cohort is large, the methods are sound,

Author response:Thank you!

but the results description is sloppy and difficult to follow for unnecessary repetition of data in the text and in the tables. The figures are useless repetitions of data already in the text and in the tables.
Author response: Thank you for your opinion. Unnecessary duplication of data in the text has been removed, while the table has been maintained. In addition, redundancies in the description of results have been eliminated.

Other points to be amended:
- Line 59: Padget is not referenced.

Author response: Thank you for your feedback. Padget's literature is not available on PubMed as it is quite outdated. In the original article, I indirectly referenced Kayembe KN's work, which discussed Padget's perspective, so I cited Kayembe KN's article. Changes have been made in Line 60 based on your suggestions.

- A schematic figure reporting anatomy and names of the circle of Willis may be useful for the non-neurologists.

Author response: Thank you for your feedback. A schematic figure illustrating the anatomy and names of the Circle of Willis has been included as Figure 2.

- Line 78-79: the sentence is unclear.

Author response: Thank you for your feedback. The sentence 78-79 has been revised in Line 80-82.
- Table 1 is better indicated as a figure.

- The imagine scheme is a recipe and not a method description.

Author response: Thank you for your feedback. Table 1 has been changed to Figure 1 in accordance with your comments.

- Line 113: hypoplasia should be better defined and its difference from dysplasia clarified.

Author response: Thank you for your feedback.In fact, the dysplasia of arteries has been defined in lines 110-111 of the original article. In this article, we do not distinguish between hypoplasia and dysplasia. In order to avoid unnecessary misunderstandings, all instances of hypoplasia have been replaced by dysplasia.
- Figure 1: name of arterial segments and indication of hypoplasia should be added with arrows.

Author response:Thank you for your feedback. We have added the names of arterial segments and indicated hypoplasia with arrows, as shown in Figure 2-3.

- Table 2: the number of subjects should be added in brackets for an easy calculation of fractions.

Author response: Thank you for your feedback.The number of subjects has been added in brackets according to your comments.

- Results sections: discuss only the significant differences, cancel useless figures and concentrate the numbers in appropriate tables.

Author response: Thank you for your feedback.We have eliminated redundant content from the Results section and condensed it within the table. 
- Tables need a heading.

Author response:All tables have a title, but when uploading the tables and the manuscript are uploaded separately, you may not see the title of the article.

- The discussion starts with an unnecessary repetition of the introduction.

Author response: The discussion section has been significantly revised based on your feedback in Line 200-210.

- Line 232: posited? Or hypothesized?

Author response:It has been revised according to your opinion, thank you!

- Line 288: I suggest “limitations”.

Author response:Thank you for your feedback.It has been revised according to your opinion, thank you!

- Reference 9 is not in a standard format.

Author response: I processed the references directly using Endnote. I have checked the names multiple times, and they are all in uppercase. Therefore, I am unsure if they are incorrect. I have made necessary changes to them.Thank you!

**Reviewer 4:**

This is a retrospective study designed to look at the association of A1 segment dysplasia on occurrence of MCA aneurysms.

Research methodology and writing are good.
Author response:Thank you!

However the manuscript does require revisions for consideration for publication. Revisions for consideration should include-

Line 41 - We cannot show that there is an effect on development or causation in a retrospective cross sectional study, I think the authors intended to use the word rupture but used development.
Author response:Thank you for your feedback.It has been revised according to your opinion, thank you!

Line 55 - What do the authors mean by " blood flow disorders". Needs elucidation.
Author response: The absence of certain Wills components hinders the normal blood flow in the circle of Willis. Changes have been made in Line 54-56.

Line 70 - We already know variations in Circle of Willis are associated with increased incidence of intracranial aneurysms. The statement should have said " incidence of middle cerebral artery aneurysms" not intracranial aneurysms in general.
Author response:It has been revised according to your opinion, thank you!

Line 89 - Please elucidate the methodology for selecting the controls.

Author response: The selection method of the control group has been described in detail as required, from Line 90 to Line 95.

Line 202 - Dysplasia as a predictor of " Rupture" not "development".
Author response:It has been revised according to your opinion, thank you!

Line 224 and 225- The reasoning for lower rate of complete Circle of Willis in the population being studied has been described but does not make sense. If age of onset of aneurysms is higher and the completeness rate of Circle of Willis is lower in younger individuals, shouldn't the completeness rate of circle of Willis be higher in this study group rather than being lower, as this is a study directed at studying patients with aneurysms who are in general older.

Author response: Thank you for your corrections. My original intention was to convey that younger people have a higher rate of Will integrity, while patients with aneurysms are typically older and thus have lower Will integrity. Changes have been made in Lines 216-220.

Line 263 - The statement regarding embryonic ICA does not make sense. This needs clarification.
Author response: Thank you for your corrections. There seems to be an issue with the translation process. I have provided further clarification and made corrections to Line 262-264.

Along with the corrections mentioned above, there seems to be predominance of ruptured aneurysm patients in this study compared to general population which usually has more patients with unruptured aneurysms. This limitation should be acknowledged.

Author response: Thank you for your opinion. This limitation has been added to the end of the article in Line 297-302.

**Reviewer 5:**

1. It appears that the A1 segment of the ACA might be associated with a smaller MCA diameter which might be associated with the formation of MCA aneurysms. However, at the end of the study, the writer listed several possible confounders to include possible selection bias.

Author response: Thank you for your opinion. You may have misunderstood my point due to the text translation. Our study shows that segment A1 of the ACA may be linked to smaller MCA diameters, which in turn may be linked to MCA aneurysm formation. However, when I mention selection bias, I am referring to the fact that we are conducting a study at a single center.

2. I am curious as to what constituted for "lack of clear image data, severe artifacts" as can be seen in line 87. This could be subjective to the radiologists or neurologists looking at the study. Or the researchers who conducted the study. This goes back to the possibility of selection bias.

Author response:What I am talking about the lack of clear image data due to significant artifacts, primarily caused by the absence of thin-layer data preservation in the system, resulting in the inability to generate 3D images during 3D-SLICER analysis. In other words, these patients underwent CTA examinations, but either the thin-layer data was not retained or only the synthesized data was saved. The thin-layer CTA data required for analysis by 3D-Slicer is missing. This issue is not related to human judgment but rather stems from the software's inability to recognize the data.
3.clearer distinction between causality and co-existence needed.

Author response:Thank you for your correction.We have made a more explicit statement in our limitation at the end of this article.

4.further distinction between the types of aneurysms may be helpful in substantiating the proposed mechanisms of such aneurysmal formation.

Author response:The aneurysms that were studied were saccular aneurysms in the brain, excluding AVM-related aneurysms and dissecting aneurysms. The inclusion and exclusion criteria have been provided in Line80-86.

**Reviewer 6:**

First, in the article the authors counted patient data spanning the corresponding CTA examinations done between 2012 and 2023, but Jiangmen Central Hospital only introduced the 64-row spiral CT described in the article in 2020, so did all the data on patients with aneurysms included in the study within the present study originate after the introduction of the 64-row CT or before? The authors should provide an explanation;

Author response:Thank you for your interest in the progress of our hospital. Our facility has actually been using the Toshiba 64-row CT scanner since 2008, and we added a Philips CT scanner in 2020. I am unsure of the source of your information indicating that our hospital would only have a 64-row CT in 2020. We have included a picture of the Toshiba CT scanner that was purchased by our hospital in 2008, but please note that the accompanying documentation is in Chinese.

Secondly, the interpretation of the CTA results was performed by a young neurosurgeon and a senior imaging physician; this arrangement reduces the accuracy of the CTA results even though both physicians independently interpreted the CTA results; two senior imaging or neurosurgeons should have performed the independent interpretation, which would have been more convincing, and the results would have been more accurate and credible. The results should be more convincing and the results more accurate and credible.

Author response:Thank you very much for your corrections. We will pay attention to this issue next time. In fact, Dr. Yue has over 5 years of clinical experience, and our team provided her with specialized training before conducting this study. I came across an example of a clinician with 2 years of experience reading radiation pictures in an article in World Neurosurgery.(1).

Once again, the authors were slightly confused when analyzing the corresponding results for comparisons between different types of subgroups; it was not clear what the p-value was when applying the statistical analysis for a statistically significant comparison between the two;

Author response:In the results section, the content has been condensed into the table to increase clarity. Additionally, the definition of the significance of the P-value has been provided.

Meanwhile, for the corresponding results of themultivariate logistic regression analysis, the results were not displayed with common graphs;

Author response:The corresponding results of the multivariate logistic regression analysis have been revised in Line185-186,193-194.
In the discussion section, the authors did not discuss and analyze the results.

Author response:We have significantly revised the discussion section by incorporating additional information on personnel screening in the first paragraph. Furthermore, we have addressed the reasons for the low integrity rate of the circle of Wills . Since the original article extensively discussed why A1 loss may result in an increase in brain aneurysms, but did not initially cite the results, there was a disconnect between the sensory content and the ensuing discussion. Thanks for your feedback, we have made corresponding adjustments to the discussion section.

**Reviewer 7:**
I would to ask you:
- how did you pair your samples, by convenience only? can you show more data (besides age and sex) so we can understand how good is the matching? have you estimated any sample size?

Author response:Pairing filtering is further elaborated on in Line90-95. Following the principle of 10 events per variable , factors such as age, gender, smoking, drinking, hypertension, diabetes, maximum length of aneurysm, and ipsilateral A1 deletion were included in this study. A sample size of more than 80 was deemed necessary for each group of data to facilitate the multi-factor logistic regression analysis. Our sample size exceeds 80 in each group.

- please add the rupture status and vasospasm occurrence in the analysis, since they are a major source of vessel diameter variation and are expected to be very different among the cases and controls?

- have you re-assessed the vessel diameter after the vasospasm phase in ruptured aneurysms?

Author response:The question you raised is very intriguing. Indeed, vasospasm resulting from ruptured bleeding can impact the diameter of the artery, and the changes observed before and after the vasospasm period following aneurysm rupture will be the focus of our subsequent research. Initially, we aimed to exclude patients who had undergone surgery at other hospitals in order to ensure the homogeneity of the images used in the study. The goal was to utilize consistent preoperative images regardless of the presence of bleeding. This was done to ensure that the condition of the blood vessels was not influenced by human intervention. 
- the vessel measurements were made on VRT images only (as suggested by Figure 1)? how did you accounted for the variability of size regarding the fitting of the opacification curves?

Author response:Many prior studies have employed planar graph research, leading to a potential issue of selection bias. In contrast, our measurement approach involved 3D state analysis, where we determined the maximum value within 1/3 of the middle section of the blood vessel under a 3D perspective. This adjustment aimed to minimize errors at the selection level.

**Reviewer 8:**

1. The purpose of this study is to explore the relationship between middle cerebral artery aneurysms and anterior cerebral arteries. What are the types of middle cerebral artery aneurysms included here? (M1 segment? bifurcation? Or M2 segment)

Author response:Our study encompassed all aneurysms within the middle cerebral artery, specifically the M1 segment, bifurcation, and M2 segment. However, in terms of incidence, bifurcation aneurysms predominate.

2. The patency of the willis loop has a significant impact on cerebral blood flow. Why is this study only focused on the relationship between the anterior and middle cerebral aneurysms in the willis? According to the author's viewpoint, the absence of other blood vessels in the Willis loop may also affect the formation of cerebral aneurysms.

Author response:You are correct. Other blood vessels in the circle of Willis can also impact the formation of aneurysms. As noted in the original article, most studies have focused on the link between dysplasia of the anterior cerebral artery and anterior communicating aneurysms, as well as the fetal posterior cerebral artery and posterior communicating aneurysms. However, the relationship between the circle of Willis and middle cerebral aneurysms remains controversial. My study aims to investigate the influence of variations in the circle of Willis on the occurrence and progression of cerebral aneurysms by examining the impact of A1 dysplasia on cerebral aneurysms.

3.In the article's methodology, it is mentioned that there is also a control group, and how the control group is selected should be detailed and reflected in the overall flowchart.

Author response:The flowchart depicting crowd inclusion has been shown in Figure1.

4.In the result part（line 204）,”The results indicated that the impact of ipsilateral A1 dysplasia on middle cerebral artery aneurysm rupture was statistically significant”. However， the purpose of the article is to confirm the impact of ipsilateral A1 dysplasia on middle cerebral artery aneurysm. The data results did not directly confirm the author's viewpoint.

Author response:We have revised the wording of the study's objective in Line70-71.

**Reviewer 9:**

Authors of the current study reported association of A1 hypoplasia with MCA aneurysm and rupture. Theoretically abnormality of intracranial vessels would affect hemodynamic flow and subsequently lead to aneurysm formation and growth. Authors proved this association with A1 hypoplasia and MCA aneurysm. I have no comments as the analysis was appropriate with appropriate statistical tools. Would recommend authors to include low power as a limitation of the study and further the results have to be validated on a larger multicenter study. Further the clinical significance of finding is missing in the paper. Would recommend authros to add this.

Author response:Thank you for your positive feedback.The clinical significance of the study has been included in the Limitations section Line301-302 at the end of the manuscript, emphasizing the need for validation in a larger multi-center study.

**References:**

1.	Zimelewicz Oberman D, Perez Akly MS, Rabelo NN, Elizondo C, Amorim Correa JL, Ajler P, et al. Morphologic Variations in the Circle of Willis as a Risk Factor for Aneurysm Rupture in the Anterior and Posterior Communicating Arteries. World Neurosurg. 2021;154:e155-62. http://doi.org/10.1016/j.wneu.2021.06.151
