# Supplementary material for: Association of middle cerebral artery aneurysms and variation of the A1 segment
Source: PLoS One. 2025 Mar 25;20(3):e0319500. doi: 10.1371/journal.pone.0319500 (PMC11936263; doi:10.1371/journal.pone.0319500)
Supplement: Attachment — Submitted filename: Response_to_Reviewers_auresp_3.docx [file pone.0319500.s003.docx]

Dear Editors,

On behalf of my co-authors, we thank you for giving us the opportunity to revise and improve the quality of our article. We have carefully reviewed the comments from both the reviewers and yourselves, and have made revisions which are marked in red within the paper. We have made every effort to address the comments provided. Please find attached the revised version, which we would like to submit for your kind consideration.

In accordance with your request, I have ensured that the citations are accurate and complete, and that no references have been made to retracted papers. Thank you.


We have prepared a detailed response letter addressing each of the reviewer comments, which can be found at the end of this letter. We found the comments to be extremely helpful and would like to express our gratitude to both you and the reviewers for your valuable feedback.

We hope that this revised version meets the standards for publication in your journal. Thank you for considering our submission, and we look forward to hearing from you soon.

 Sincerely,

Hongzhi Gao

**Reviewer #1:** The authors have made the necessary changes and the manuscript can be accepted in its current format for publication.
Author response:Thank you！

**Reviewer #2:** The objective of this paper is commendable，it utilizes clinical data for publication and possesses the necessary ethical approval documents, making it suitable for dissemination.

Author response:Thank you！

**Reviewer #3:** The authors have accepted all the comments and have implemented the necessary corrections. However, there are still inconsistencies and errors that need a careful revision by an English-speaking scientific expert.
They can be enlisted as follows:
- Line 115, a verb is missing.

Author response:Thank you for the correction. We have added the verb.

- Figures 4 and 6 captions are awkward.
- Heading of table 1 is too descriptive.
- Description of column 1 in table 1 need rewording.
- Heading of table 2, 3, and 4 is too descriptive. These comments should stay in the text.

Author response:Thank you for your corrections, we have revised the manuscript according to your suggestions.

- There is no consistency between abbreviations in fig. 2, 3 and text.

- Letters of P2 and MCA in figure 2 are too clear to be recognized.

Author response:Thank you for your comment. I have redrawn a clearer Figure 2 and added annotations for the abbreviations.

- The sentence on line 220 is unclear.

Author response:Thank you for the correction. I have reorganized the sentence in line 220 to make it more coherent.

**Reviewer #6:** The author responded to each of the corresponding questions I asked, but two of them were not convincingly answered. Therefore, I believe that the article needs to be further revised to meet the criteria for publication in your journal.

Author response:Thank you very much for your constructive criticism. However, I am sorry to say that you did not specify which two issues did not convince you, so I am unable to make further revisions. Thank you!

**Reviewer #7:** Dear Authors

I read the revised version of the manuscript. You addressed some issues; core questions remain, however:
1) This is a cross-sectional study. Therefore, it is not scientifically possible to establish a temporal correlation between "dysplasia" and "aneurysm development". So, make your work consistent with the methodology and change your writing to "correlated", "associated", and the like.
Author response:Thank you for your comment. This retrospective study begins with two groups, one with the middle cerebral artery aneurysms (cases) and one without (controls). Then we look back in time to assess exposure history in both groups.So we believe our study is a case-control study, which allows us to assess a possible causal relationship between the disease and exposure factors.

2) The occurrence of subarachnoid hemorrhage is at the core of your investigation! Seventy-two percent of the aneurysm group had it. Nearly half of these patients will have temporarily false measures of the vessels' diameter - A1 included. Since you have not made a follow-up imaging, you have two ways to solve this: a. include the ruptured status in the multivariate analysis and discuss accordingly. Prevalence-wise, your findings will likely correlate to ruptured status. Then, discuss how this impacts the results. Or b. discuss that your methodology is insufficient to provide clear conclusions about baseline vessel assessments - therefore, you cannot exclude that the results can be biased. Accordingly, your conclusion should be that this exploratory study generates the hypothesis that longstanding A1 changes could correlate to MCA aneurysms, which should be the subject of a subsequent, more robust investigation.
   Author response:Thank you for your comment. In fact, I have already discussed the relevant issues in lines 302-305 of the Limitation section. We have also made revisions to the conclusion section based on your suggestions.

3) The prevalence of A1 dysplasia in the aneurysm group was 39/269 (0.1449). The prevalence of A1 dysplasia in the control group was 29/269 (0.1078). The reported p-value (P = 0.0125) is not reproducible - neither by the two-sided Fisher nor the Chi-square tests. I found the significance levels varied from 0.2428 to 0.1945. For the suggested level of significance, you would need at least 1,263 subjects. If this is not the case, please explain and attach you statistical calculations to clarify.

Author response:Thank you very much for pointing out our significant mistake. We have rechecked the data and found that 20 was mistakenly written as 29. You can roughly estimate from the bar chart in Figure 7 that our data should be 20 instead of 29. The data in the manuscript has been corrected. We sincerely hope that you can forgive us for making such a basic error. Thank you!

3) You replied about the measurement methodology question, and cited two articles. The cited studies does not backup your claims. If you take a closer look at the Krasny (DOI:10.1136/neurintsurg-2013-010669) and Lazzaro (DOI:10.1136/jnis.2010.004358), both used planar DSA as the reference method. Interestingly, those authors address the limitations of CTA in assessing the vessels diameters. My suggestions are: a. find the appropriate references that back up your claims, but be mindful that 95% of reader are convinced that DSA is still the gold standard, as medication societies support. Or b. state that you developed this methodology and discuss the limitations.
   Author response:Thank you for your comment.Allow me to explain this issue. The controversy between DSA and CTA in assessing vascular accuracy has been ongoing for a long time, with DSA being considered the gold standard. However, in clinical practice, there are many cases where there is significant external vessel diameter thickening and internal lumen narrowing. Our understanding is that DSA more accurately assesses the internal diameter of vessels, while CTA can more accurately assess the external diameter of vessels. In studying the integrity of the Wills' circle, the disadvantage of DSA is that it cannot simultaneously visualize all vessels of the circle and may not visualize vessels that actually exist but have very slow flow. Therefore, some scholars tend to use CTA to study the integrity of the Wills' circle, as mentioned in lines 215-216 of the original manuscript. We also believe in the authority of DSA, but in studying changes in vessel diameter, CTA can achieve results similar to DSA. However, we consider this is not the main focus of this article, so we only briefly touch upon it. Regarding the citation you mentioned, I borrowed the measurement method in the mid-segment 1/3 of the vessels from these two authors. Thank you!

**Reviewer #9:** (No Response)

Author response:Thank you!
