# Supplementary material for: Association of middle cerebral artery aneurysms and variation of the A1 segment
Source: PLoS One. 2025 Mar 25;20(3):e0319500. doi: 10.1371/journal.pone.0319500 (PMC11936263; doi:10.1371/journal.pone.0319500)
Supplement: Attachment — Submitted filename: Response_to_Reviewers_auresp_4.docx [file pone.0319500.s004.docx]

Dear Editors,

On behalf of my co-authors, we thank you for giving us the opportunity to revise and improve the quality of our article. We have carefully reviewed the comments from both the reviewers and yourselves, and have made revisions which are marked in red within the paper. We have made every effort to address the comments provided. Please find attached the revised version, which we would like to submit for your kind consideration.

In accordance with your request, I have ensured that the citations are accurate and complete, and that no references have been made to retracted papers. Thank you.


We have prepared a detailed response letter addressing each of the reviewer comments, which can be found at the end of this letter. We found the comments to be extremely helpful and would like to express our gratitude to both you and the reviewers for your valuable feedback.

We hope that this revised version meets the standards for publication in your journal. Thank you for considering our submission, and we look forward to hearing from you soon.

 Sincerely,

Hongzhi Gao

Reviewer #3: The authors have revised most of the comments of the reviewers, but the paper is still difficult to read for grammar and English style errors, for inconsistencies e needless repetitions.
To mention a few:
- In figures 4 to 8 the order of the columns is always different (control and aneurisms).

Author response:Thank you for your comments. Figure 4-8 has been modified according to your comments.

- M1 in figure 2 is not indicated
- The aneurism in figure 3 is indicated in the caption but not in the image.

Author response:In fact, Figure 2 of the original image shows the MCA, which is actually M1 and has been modified according to your opinion. The aneurysm in Figure 3 is represented with a double arrow.Thank you!

- The short title is as long as the full title.

Author response:Thank you for your comments, which have been revised according to your comments.

- “Fetal” and “embryonic” are used in an unclear way.

Author response:It has been modified according to your opinion, thank you！

- Line 60: reference to Padget is not accompanied by a correct citation in the reference list at number 16.

Author response:The original reference 16 refers to an early document where the information is actually found in reference 17. In my initial draft, I cited reference 17, but some experts suggested citing reference 16 instead. However, I couldn't locate a standardized format for this citation. Ultimately, I determined that whether this reference was included or not did not significantly alter the intended meaning of the full text, so I opted to remove this information.

- Line 98: The imaging scheme is still a kind of radiological recipe, unsuitable for the methods section.

Author response:The imaging scheme has been deleted according to your opinion, and the imaging scheme has been added to the supplementary materials.

- Line 104: tracker tracking?

Author response:It has been revised according to your comments. Thank you!

- Line 119: calculated or rather counted?

Author response:It has been revised according to your comments. Thank you!

- Line 124: A1 vessel measurement is not visible. The left and right sides should also be indicated.

Author response:It has been revised according to your comments. Thank you!

- The headings of the tables are at the bottom and not above the table.

Author response:It has been revised according to your comments. Thank you!

- Line 155: what is the development?

Author response:It has been revised according to your comments. Thank you!

- Table 1: the rows are unclear as are their titles. Some significances are missing in the right column.
Author response:It has been revised according to your comments. Thank you!

- Line 181: the diameters are not reported and there is no reference to table 2 where it is described.

Author response:Due to previous reviewer feedback indicating redundancy between the main text and the tables, some content has been streamlined. The table now showcases only the most valuable information, with additional details presented in the main text.

- Table 4: the headings of the first column are in multiple lines and comprehension of the table is difficult.

Author response:It has been revised according to your comments. Thank you!

- Line 208: researchs should be research.

Author response:It has been revised according to your comments. Thank you!

- Line 235: Reference 17 is missing after Kayembe and reported in the following sentence, which refers to a different study.

Author response:I think it was deleted by mistake. I have added it. Thank you!

- Line 287: the highest.

Author response:It has been revised according to your comments. Thank you!

- Line 309: And…

Author response:It has been revised according to your comments. Thank you!

Reviewer #6: After reading the revised manuscript, I have the following suggestions: In terms of the current mainstream view, the vast majority of physicians firmly believe that digital subtraction angiography (DSA) is still the gold standard for the diagnosis of cerebrovascular disease. This is especially true for issues such as cerebrovascular diameter; however, all of the data in this article are derived from CTA results, and it is recommended that this controversial issue and the limitations of CTA be discussed.

Author response:Thank you for your comment.Allow me to explain this issue. The controversy between DSA and CTA in assessing vascular accuracy has been ongoing for a long time, with DSA being considered the gold standard. However, in clinical practice, there are many cases where there is significant external vessel diameter thickening and internal lumen narrowing. Our understanding is that DSA more accurately assesses the internal diameter of vessels, while CTA can more accurately assess the external diameter of vessels. In studying the integrity of the Wills' circle, the disadvantage of DSA is that it cannot simultaneously visualize all vessels of the circle and may not visualize vessels that actually exist but have very slow flow. Therefore, some scholars tend to use CTA to study the integrity of the Wills' circle, as mentioned in lines 215-216 of the original manuscript. We also believe in the authority of DSA, but in studying changes in vessel diameter, CTA can achieve results similar to DSA. However, we consider this is not the main focus of this article, so we only briefly touch upon it.
